# Analysis of the Genetic Diversity and Family Structure of the Licha Black Pig Population on Jiaodong Peninsula, Shandong Province, China

**DOI:** 10.3390/ani12081045

**Published:** 2022-04-17

**Authors:** Yuan Wang, Ruilan Dong, Xiao Li, Chao Cui, Guanghui Yu

**Affiliations:** 1 College of Animal Science and Technology, Qingdao Agricultural University, Qingdao 266109, China; wangyuan@qau.edu.cn (Y.W.); dongrl@qau.edu.cn (R.D.); lixiaoful@163.com (X.L.); 2 Bureau of Agriculture and Rural Affairs of Jiaozhou, Jiaozhou 266300, China; 13869809693@163.com

**Keywords:** Licha black pig, single nucleotide polymorphism, genetic diversity, population structure, inbreeding coefficient

## Abstract

**Simple Summary:**

This study investigated the current conservation status, including the genetic diversity, the family structure, and inbreeding, of the Licha black pig population on Jiaodong Peninsula (Shandong Province, China). The results provide insights into the management and conservation of a local pig breed. Breeders are encouraged to utilize genomic data to improve mating schemes based on the family information obtained in this study, such as keeping an equivalent number of boars and sows in each family and selecting individuals with a kinship coefficient of less than 0.1 for mating.

**Abstract:**

The Licha black pig, a popular indigenous Chinese pig breed, is known for its multi-vertebral trait and higher lean meat rate. Understanding the current conservation status, family structure, and degree of inbreeding of the Licha black pig population will be useful to maintain a sufficient level of genetic diversity in these animal resources. In the present study, the genetic diversity, population structure, and inbreeding coefficient of this conserved population were analyzed using SNP genotyping data from 209 Licha black pigs. Based on the genomic information, this population was divided into eight different families with boars. The effective population size (N_e_), polymorphic marker ratio (P_N_), expected heterozygosity (H_e_), and observed heterozygosity (H_o_) of this population were 8.7, 0.827, 0.3576, and 0.3512, respectively. In addition, a total of 5976 runs of homozygosity (ROHs) were identified, and most of the ROHs (54.9%) were greater than 5 Mb. The genomic inbreeding coefficient of each individual was estimated based on ROHs (*F*_ROH_) with an average inbreeding coefficient of 0.11 for the population. Five statistics (N_e_, P_N_, H_o_, H_e_, and *F*_ROH_) showed a decrease in the level of genetic diversity and a high degree of inbreeding in this population. Thus, special preservation programs need to be implemented in the future, such as introducing new individuals or improving the mating plan. Altogether, our study provides the first genomic overview of the genetic diversity and population structure of Licha black pigs, which will be useful for the management and long-term preservation of this breed.

## 1. Introduction

China has an abundance of genetic resources for pig breeds and hosts almost one-third of all pig breeds in the world [1]. Many indigenous pig breeds have their own unique characteristics, such as disease resistance, feed efficiency, and a high fertility rate [2]. However, due to the introduction of commercial pig breeds, the genetic resources of indigenous pig breeds have become endangered, and both the number of local breeds and the population size have decreased dramatically [1]. Therefore, it is necessary to assess the genetic diversity and population structure of local Chinese pig breeds for the development of gene-based selection tools and breed conservation. In the past, traditional pedigree records were used in population structure analyses, but these may be affected by incomplete parental information or incorrect pedigree records [3,4]. With the development of sequencing technology, genetic relationships between individuals could be constructed using single nucleotide polymorphism (SNP) chips or re-sequencing data [5], which can help improve breeding and conservation programs. In recent years, the genetic status of some indigenous pig breeds, such as Liangshan pigs [6], Laiwu pigs [7], Ningxiang pigs [8], and Jiangquhai pigs [9], has been studied. These results are beneficial for research on pig genetic resources (Supplementary Table S1).

The Licha black pig is a popular indigenous Chinese pig breed that is mainly distributed on Jiaodong Peninsula (Shandong Province, China) and has a long history of more than 2000 years. Compared with other local breeds, it is known for its multi-vertebral trait, which has one or two additional thoracic and lumbar vertebrae [10]. Selecting multi-vertebral individuals will further increase pork production and economic benefits. However, little is known about the current conservation status, potential risk of inbreeding depression, and family structure of the Licha black pig population. Thus, it is essential to study the genetic diversity and population structure of Licha black pigs to maintain a sufficient level of genetic diversity in these animal resources.

In this study, we selected a 50 K SNP chip to analyze the genetic diversity, genetic relationships, population structure, and inbreeding coefficient in this Licha black pig population. The results may be useful for the management and preservation of this breed.

## 2. Materials and Methods

### 2.1. Animals

A total of 209 individuals, including 30 boars and 179 sows, from the National Nucleus Licha Black Pig Conservation Farm in Jiaozhou, Shandong Province were used in this study. In the current breeding process, a closed-nucleus breeding scheme is used in this population. Boars were selected based on their physical appearance, growth rate, and body size, and random mating occurred between boars and sows. In this study, all the animals were sampled within a restricted time period. Ear tissues were collected using ear scissors and placed in a centrifuge tube containing anhydrous ethanol.

### 2.2. Genotyping and Quality Control

Genomic DNA was extracted from ear tissues using TIANamp Genomic DNA kits (Tiangen Biotech, Beijing, China). The concentration and purity of genomic DNA were assessed using a NanoDrop™ 2000 (Thermo Fisher, Waltham, MA, USA), and all DNA samples with a ratio of light absorption (A260/280) between 1.8 and 2.0 and a concentration >50 ng/μL were eligible for genotyping. Individual genotyping was conducted using the “Zhongxin-I” Porcine Chip (Beijing Compass Agritechnology Co., Ltd., Beijing, China), which contains 51,315 SNPs across 18 autosomes and 2 sex chromosomes. PLINK software (v1.90) [11] was used for quality control.

We excluded 1180 SNPs with call rates less than 90%, 4550 SNPs with minor allele frequencies less than 0.01, 607 SNPs with Hardy–Weinberg equilibrium *p* values less than 1 × 10^−6^, and 6576 SNPs located on sex chromosomes. Finally, a total of 209 individuals and 38,402 autosomal SNPs were retained for subsequent analysis.

### 2.3. Genetic Diversity Analysis

Effective population size (N_e_), polymorphic marker ratio (P_N_), expected heterozygosity (H_e_), and observed heterozygosity (H_o_) are widely used parameters in the analysis of the genetic diversity of populations.

N_e_ is estimated based on the level of linkage disequilibrium. The equation $N_{e}=\frac{1}{4c}\times \left(\frac{1}{r^{2}}-1\right)$ was used to calculate N_e_, where c is the genetic distance between two SNPs expressed in Morgans and *r*^2^ is the LD of different distances [12]. In this study, SNeP software was used to compute N_e_, which was also corrected with the sample size [13].

P_N_ is the ratio of polymorphic sites to the total number of sites. Firstly, we used PLINK software (v1.90) to calculate the minimum allele frequency of each site, and then used the following formula to calculate P_N_:

$$P_{N}=\frac{M}{N},$$

where M is the number of sites exhibiting polymorphisms and N is the total number of sites.

H_e_ is the ratio of heterozygosity at any site across all individuals, and H_o_ is the ratio of individuals where a site is heterozygous compared to all individuals. If H_e_ is less than H_o_, the population may be influenced by migration or gene flow, while if H_e_ is more than H_o_, selection or inbreeding may have occurred in the population [6,14]. PLINK software (v1.90) was used to calculate He and H_o_.

### 2.4. Genetic Relationships and Population Structure Anaylsis

As compared with pedigree-based relationships, a genomic relationship matrix constructed using 38,402 autosomal SNP markers can better reflect the true kinship between individuals. G matrix (V2) was used to calculate kinship values, and heat maps were used to visualize the results on kinship [15]. In addition, PLINK software (v1.90) was used to construct an identity-by-state (IBS) distance matrix. Based on the IBS distance matrix, the population structure was clustered using the neighbor-joining (NJ) method and visualized using Mega X software [16]. By combining genetic relationship and population structure analyses, we can roughly judge which Licha black pigs were related and likely originated from the same familial lineages.

### 2.5. Inbreeding Coefficient Analysis

Runs of homozygosity (ROHs) are adjacent segments of the genome in which two haplotypes inherited from the parents are homologous [3,17]. The length and frequency of ROHs can reflect the group history. Longer haplotypes indicate a recent genetic relationship, while shorter haplotypes indicate an ancient inbreeding relationship [17]. PLINK software (v1.90) was used to identify ROHs in each sample. We set the following criteria to define a ROH using the *-homozyg* option: (1) a minimum of 30 consecutive SNPs included in a ROH; (2) a sliding window with fifty SNPs that moves one SNP at a time; (3) a minimum density of one SNP in 1000 kb; (4) a maximum of one missing genotype and one heterozygous genotype in a ROH; (5) a window threshold of 0.05; (6) a minimum ROH length of 1 Mb; and (7) a maximum gap between consecutive SNPs of 1 Mb. Furthermore, we divided the ROHs into three types: 1~5, 5~10, and >10 Mb [18].

Genomic inbreeding coefficients were estimated based on ROHs (*F*_ROH_), and the *F*_ROH_ for each individual was calculated using the following equation:

$$F_{\mathrm{ROH}}=\frac{\sum_{i}L_{\mathrm{ROH}i}}{L_{\mathrm{auto}}},$$

where $L_{\mathrm{ROH}i}$ is the length of the ROH in the individual and $L_{\mathrm{auto}}$ is the autosomal genome length of the species (Sscrofa 10.2 reference genome assembly; the autosomal length is approximately 2,450,713 kb).

## 3. Results

### 3.1. Genetic Diversity of Licha Black Pigs

The N_e_ of the 209 individuals from the Licha black pig conservation farm was 8.7, and the ratio of P_N_ was 0.827. Here, H_o_ (0.3576) was greater than H_e_ (0.3512), which indicated that the population may have been influenced by migration or gene flow.

### 3.2. Genetic Relationships between and Population Structure of Licha Black Pigs

**Figure 1:**
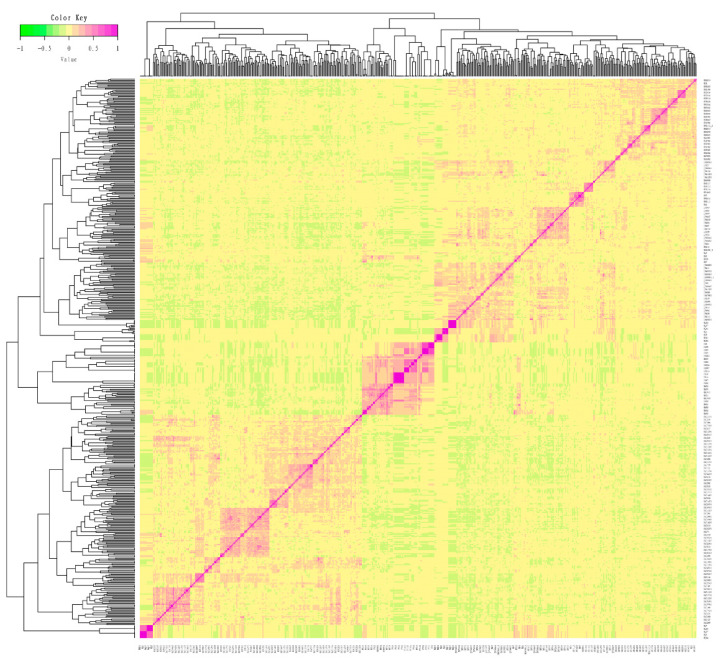
***G*** matrix heat map of Licha black pigs in the conserved population. Each small square exhibits the kinship value between different individuals. The closer the color of squares is to red, the closer the kinship between individuals.

In this conserved population, the genetic relationship coefficients ranged from −0.22 to 0.89 (Supplementary Table S2), and the visualization results of the ***G*** matrix are exhibited in Figure 1. Among these relationships, some individuals have closer genetic relationships, while most of them have moderately close genetic relationships.

**Figure 2:**
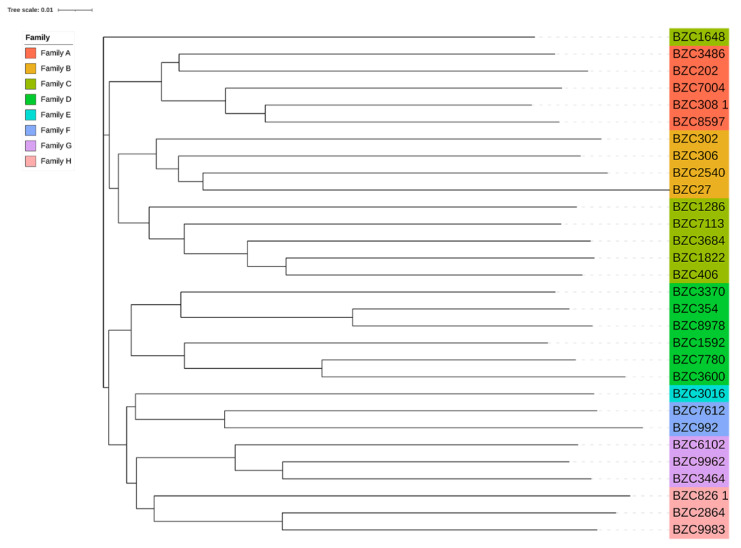
Phylogenetic tree of boars in this population. Individuals with the same color belong to the same familial lineage.

**Figure 3:**
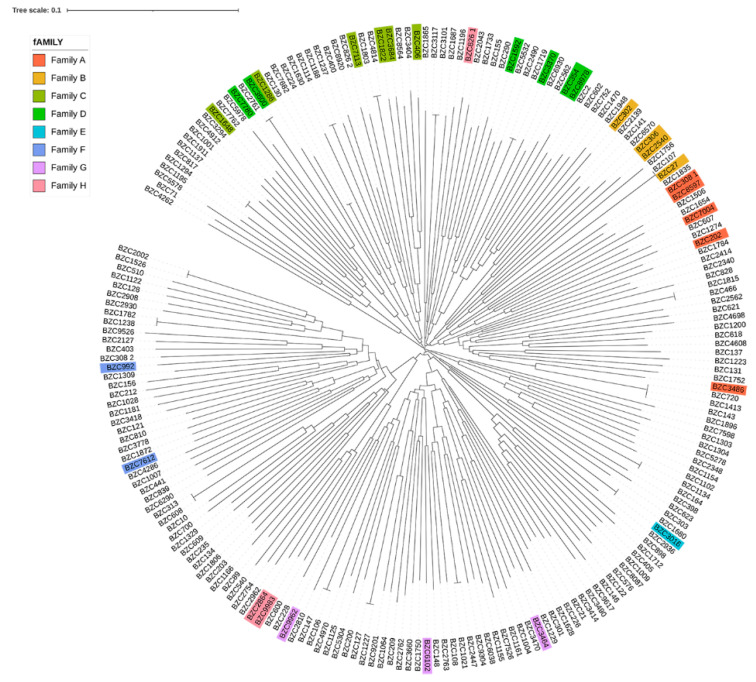
Phylogenetic tree of all individuals in this population. Individuals with the same color belong to the same familial lineage.

Considering the importance of boars to the breeding process, we analyzed the phylogenetic tree of boars by the NJ method. As shown in Figure 2, the phylogenetic tree divided the 30 boars into eight different familial lineages of similar genetic ancestors. Individuals with the same color belonged to the same familial lineage, and the branches for the same lineage were divided into different inner clustering units. Furthermore, based on the genomic relationship between boars and sows, these individuals were classified into eight large families (Figure 3). In addition, two sows were distantly related to the 30 boars (the genetic relationship coefficient was less than 0.1), and they were classified as another family (Supplementary Table S3).

### 3.3. Inbreeding Coefficient of Licha Black Pigs

In total, 5976 ROHs were identified by PLINK software (v1.90) on the 18 autosomes in the Licha black pig population (Supplementary Table S4), with an average number of ROHs per individual of 28.59. The length of each ROH ranged from 1.64 Mb to 145.46 Mb, and the classification statistics of ROHs are summarized in Table 1. Most ROHs were 1~5 Mb in length; however, these ROHs covered a relatively small proportion of the genome compared with other types.

**Figure 4:**
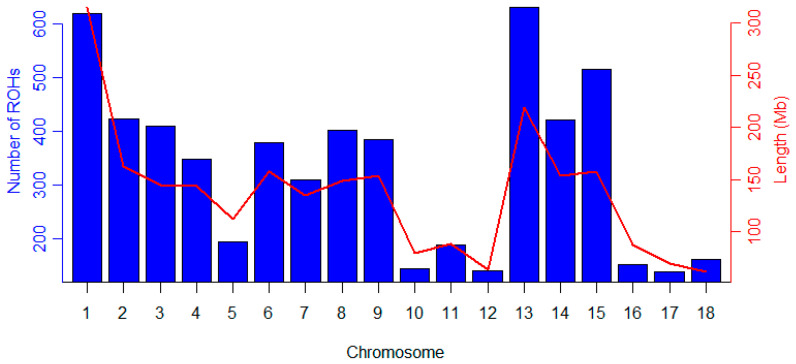
Frequency distribution of ROHs per chromosome (blue bars) and changes in the length of each chromosome (red lines).

**Figure 5:**
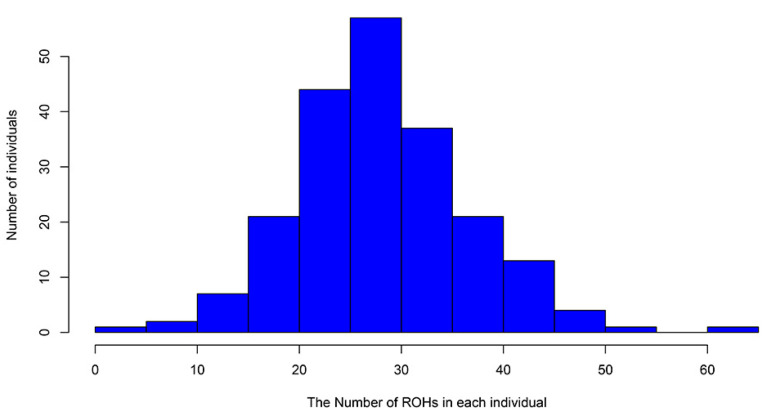
Frequency distribution of ROHs in each individual.

As shown in Figure 4, the number of ROHs on autosomes is related to the increase in the chromosome length; the number of ROHs on each chromosome was the largest on SSC 13 (632), whereas the number of ROHs on each chromosome was the smallest on SSC 17 (139). The total number of ROHs in each individual varied from 3 to 62, with an average of 28.59 ± 8.74 (Supplementary Table S5), and most individuals had 25 to 30 ROHs (Figure 5). In this population, *F*_ROH_ for each individual was calculated from the detected ROHs (Supplementary Table S5), and the average inbreeding coefficient of this population was 0.11 ± 0.05.

## 4. Discussion

The Licha black pig is a popular local Chinese pig breed. Genetic diversity and population structure analyses based on genetic data can improve or support selection towards the maintenance of genetic diversity. In the present study, the “Zhongxin-I” Porcine Breeding Chip was used to conduct individual genotyping, as this chip included the unique SNPs of local breeds and the important functional candidate gene sites. The application of this chip will provide an effective approach to indigenous pig breeding and improve the accuracy of population parameter estimation [19].

The N_e_ of the Licha black pig population was 8.7, which is lower than that of other local pig breeds in China [6,20]. We considered that the reason for this result may be the limited population size, the high degree of inbreeding, and the closed nucleus breeding scheme, which has led to a decreased level of genetic diversity in this population. Thus, in the conservation of Licha black pigs, we need to develop mating schemes to prevent the loss of independent familial lineages and to increase genetic exchange by introducing new individuals from the provincial nucleus Licha black pig conservation farm. The construction of a new conservation farm by collecting individuals raised by farmers may also enrich the genetic diversity of the Licha black pig breed. The P_N_ in this population was 0.827, which is also less than that of other local breeds, such as Jinhua, Erhualian, Kele, and Rongchang pigs [5,21]. This variation may be due to the difference between the sample sizes and the algorithms used. Heterozygosity is considered to be a useful parameter in estimating the genetic diversity of populations [22]. In this population, H_o_ was greater than H_e_, indicating that some Licha black pigs may have crossbred with other pig breeds before the construction of this conservation farm. Further homozygous mating between these individuals needs to be conducted by analyzing the familial lineages of Licha black pigs in the future. In addition, compared with previous studies, the heterozygosity (both H_e_ and H_o_) in the Licha black pig population in this study was lower, which may have been caused by inbreeding or the loss of families with lower productivity [3,6].

The accuracy and completeness of pedigrees play key roles in the breeding process. However, data recording errors are inevitable at local pig conservation farms, and the average pedigree error rate can reach 10% [23]. A genomic relationship matrix can reflect the kinship between individuals, which can be used to help correct pedigree errors and effectively protect the Licha black pig population. We also collected individuals with both pedigree records and SNP genotyping data and found that the accuracy of the existing pedigree records was poor (Supplementary Table S6), which also illustrates the importance of research on the division of familial lineages. In this study, the Licha black pig population was classified into eight different familial lineages with boars. Among them, the number of boars in four families was less than four. Therefore, to maintain a balanced family structure, breeders are encouraged to focus on the selection of the number of boars per family in subsequent conservation work. Furthermore, the phylogenetic tree of all individuals can be used as a reference to establish frozen semen banks of different families, which will improve the conservation efficiency of the Licha black pig population.

Traditionally, inbreeding coefficients have been calculated using the pedigree records. As mentioned above, pedigree information might be incomplete, while genomic information can provide a more accurate description of the relationship than pedigrees, as the inbreeding coefficient can also be calculated [24]. Bosse et al. [25] found that ROH lengths greater than 5 Mb were as accurate as genome sequencing. In this study, 54.9% of the ROHs were greater than 5 Mb, which indicates the accuracy of ROH detection using medium-density SNP chips. However, SNP chip data did not cover all the loci on the genome, which may cause an ascertainment bias in the analysis of genetic diversity. Thus, re-sequencing data will be generated and analyzed for this breed in the future, especially for the genetic mechanism of the multi-vertebral trait. In addition, the length of ROHs can reflect the time that inbreeding occurred, and the proportion of ROHs with lengths longer than 10 Mb among ROHs of all lengths was the highest at 60.06%. These long ROHs are the result of recent inbreeding [26]. Based on genomic information, *F*_ROH_ might be a more accurate alternative for estimating animal relatedness and inbreeding levels in theory [27]. We calculated the *F*_ROH_ of each individual, and the average inbreeding coefficient of the whole conserved population was 0.11, indicating that a high degree of inbreeding occurred in this population. For highly inbred individuals, breeders need to focus on the potential risk of inbreeding depression. Compared with other local breeds in China, the inbreeding coefficient of the Licha black pig population was higher than that of the Liangshan pig population [6], but lower than that of the Laiwu, Ningxiang, and Wannan black pig populations [7,8,28]. Both the limited population size and the relatively closed operation system may have affected the level of inbreeding. Thus, in order to reduce the potential for inbreeding depression, special preservation programs should be implemented, such as keeping an equivalent number of boars and sows in each family and selecting individuals with a kinship coefficient of less than 0.1 for mating. Additionally, the results of this study may also provide a reference for the conservation of other local breeds in the future.

## 5. Conclusions

This study represents the first genetic survey of the genetic diversity and population structure of the Licha black pig population. Five statistics (N_e_, P_N_, H_o_, H_e_, and *F*_ROH_) collectively show that we need to increase the level of genetic diversity in the current population and mitigate the potential risk of inbreeding depression. Furthermore, the obtained genomic family information can better exhibit the kinship between individuals and provide a theoretical basis for making mating plans.

## Figures and Tables

**Table 1:** Descriptive statistics of the three classes of ROHs.

Type of ROH	ROH Counts	Number Percentage (%)	Mean ± SD (Mb)	Total ROH Length (Mb)	Length Percentage (%)
ROH 1~5 Mb	2695	45.1	3.63 ± 0.73	9786.74	17.35
ROH 5~10 Mb	1832	30.66	6.96 ± 1.38	12,755.55	22.62
ROH > 10 Mb	1449	24.24	23.36 ± 17.5	33,851.14	60.03

## Data Availability

The genotype data on the samples used in the present study are available from the FigShare Repository: https://figshare.com/articles/dataset/Analysis_of_genetic_diversity_and_population_structure_of_the_Licha_black_pig_population/15170826 (accessed on 16 August 2021).

## Supplementary Materials

The following supporting information can be downloaded at: https://www.mdpi.com/article/10.3390/ani12081045/s1. Table S1: Literature review of the genetic diversity in pigs using SNP chip data; Table S2: Genetic relationship coefficients matrix; Table S3: Population structure of all individuals; Table S4: Information on identified ROHs; Table S5: Inbreeding coefficient value of each individual; Table S6: Comparison between pedigree information and genetic relationship coefficients.
